# The impact of the pandemic on mothers and children, with a focus on syndemic effects on black families: the “Prenatal to Preschool” study protocol

**DOI:** 10.3389/fpsyt.2023.1281666

**Published:** 2024-01-08

**Authors:** Wanjikũ F. M. Njoroge, Tiffany Tieu, Devlin Eckardt, Megan Himes, Christina Alexandre, Waynitra Hall, Kate Wisniewski, Ayomide Popoola, Kayla Holloway, Yuheiry Rodriguez, Sara Kornfield, Florence Momplaisir, Xi Wang, Raquel Gur, Rebecca Waller

**Affiliations:** ^1^Perelman School of Medicine, Department of Psychiatry, University of Pennsylvania, Philadelphia, PA, United States; ^2^Department of Child and Adolescent Psychiatry and Behavioral Sciences, Children’s Hospital of Philadelphia, Philadelphia, PA, United States; ^3^PolicyLab, Children’s Hospital of Philadelphia, Philadelphia, PA, United States; ^4^Lifespan Brain Institute (LiBI), Children’s Hospital of Philadelphia and Penn Medicine, University of Pennsylvania, Philadelphia, PA, United States; ^5^Clinical Research Support Office, Children’s Hospital of Philadelphia, Philadelphia, PA, United States; ^6^Department of Neuroscience, University of Pennsylvania, Philadelphia, PA, United States; ^7^Department of Psychology, University of Pennsylvania, Philadelphia, PA, United States; ^8^Penn Center for Women’s Behavioral Wellness, Department of Psychiatry, Perelman School of Medicine, University of Pennsylvania, Philadelphia, PA, United States; ^9^Perelman School of Medicine, Division of Infectious Diseases, University of Pennsylvania, Philadelphia, PA, United States

**Keywords:** racial and ethnic disparities, social determinants of health, child, infant, preschool

## Abstract

**Introduction:**

Racism, a known social determinant of health, affects the mental health and well-being of pregnant and postpartum women and their children. Convincing evidence highlights the urgent need to better identify the mechanisms and the ways in which young children’s development and mental health are adversely impacted by their mothers’ experiences of racism. With the additional stressor of the COVID-19 pandemic, the criticality of improving knowledge of these domains has never been starker. The proposed project will address these questions and move the field forward to create targeted, culturally informed preventative interventions, thus achieving mental health equity for all children and families.

**Methods:**

This prospective research is a cohort study that will longitudinally observe the outcomes of a cohort of mothers and their children recruited during the initial phase of the COVID-19 pandemic. Data will be parent/caregiver questionnaires assessing mental health, racism, support, and resilience at multiple time points with the first beginning at 24 months, clinical interviews with mothers, electronic medical records of mothers, and videotaped dyadic interactions at child age 24 and 48 months. A subset of Black participants will be asked to participate in qualitative interviews at child age 36 months.

**Results:**

Analyze will be performed within and across Black and Non-Latino/a/e/x white (NLW) groups, and comparing mothers and fathers/secondary caregivers. Descriptive and multivariate analyzes will be run to better characterize how young children’s development and mental health may be adversely impacted by their caregiver’s experiences of racism.

**Discussion:**

This prospective longitudinal mixed-methods study evaluates the simultaneous effects of the COVID-19 pandemic and racism on mothers and their developing children to characterize cross-racial differences, providing insight into risk and resilience factors in early development and the peripartum period.

## Introduction

The COVID-19 pandemic had an overwhelming and disproportionate impact on Black, Indigenous, and Latino/a/e/x communities in the US (1), including higher rates of infection (2), mortality (3), unemployment (4), housing and food insecurity (3), and adverse mental health outcomes (5). The COVID-19 pandemic precipitated worsening mental health among parents and children (6, 7) though few studies have investigated longer-term effects among Black, Indigenous, and Latino/a/e/x parents of very young children (8). Lack of social support, adverse mental health experiences, or major/chronic environmental stressors within families or communities (i.e., racism and/or a global pandemic) are known to negatively impact caregiving practices (9), the parent–child relationship (10, 11), and child brain development (12), with lasting implications for children’s mental health (13). These factors have downstream effects on children via their parents and environmental influences (the “exposome”), such as intrauterine conditions, poverty, racism, psychosocial stressors, and traumatic life events, which confer significant neurodevelopmental risk (14). However, few studies have provided rich qualitative *and* quantitative characterization of the impact of the prenatal exposome on infant and early childhood development and mental health (15, 16).

### Syndemic framework

Few studies have investigated the impact of the syndemic on Black families compared to non-Latino/a/e/x white (NLW) families. Here, we refer to the intersection of systemic racist conditions and the COVID-19 pandemic as a syndemic, *defined as the aggregation of two or more endemic and epidemic conditions leading to adverse repercussions for health* (17). The syndemic framework encapsulates the burden of the COVID-19 pandemic, which compounded underlying structural racism embedded within families’ experiences of healthcare, housing, employment, and education for Black, Indigenous, and Latino/a/e/x families, widening known health disparities (18). No studies have examined the impact of the syndemic on the mental health and developmental outcomes of Black mothers and children when compared to NLW mothers and children.

Neuroscientific advances over the past couple of decades have highlighted the importance of understanding the impact of multiple early adverse exposures on developing children beginning *in utero* and continuing through the first 5 years of life (19). Recent additional studies of structural racism and other stressors disproportionately experienced by Black children and their caregivers during sensitive developmental periods expand these findings (20). Thus, the effects of the syndemic may be most pernicious for very young children as 90% of brain development occurs during the first 5 years of life, making this period more vulnerable to disruption (21). This study investigates how syndemic stress impacts the mental health of mothers, recruited while pregnant at the start of the pandemic in the US, and that of their children. In addition, this study includes a qualitative assessment that will promote a more detailed characterization of the specific experiences of Black mothers. Examining critical developmental periods and protective parenting factors is key to the future development of precise and culturally informed interventions.

### Racism and early child development

The most recent U.S. census data reflects rapidly evolving racial/ethnic demographics. The majority of the 75 million children under the age of 15 are now children of color, outnumbering NLW children (22). Data from the 2021 Children’s Defense Fund also reports that nationally, 51% of children are children of color (23). Despite the marked diversifying of the nation in the pediatric population, there is a profound lack of recognition of the current realities faced by many Black, Indigenous, and Latino/a/e/x children, and the ways in which structural racism impacts development (24–26). Here, we define structural racism as “*practices that maintain or exacerbate unfair inequalities in power, resources or opportunities across racial, ethnic, cultural or religious groups”* (27). Racism has immediate and lasting effects on the developing brain, including via stress-related epigenetic changes (28, 29) and in the experience of chronic stress and historical trauma of mothers that may in turn, shape the experiences of their children (30–32). In light of growing evidence from studies that have documented dramatic consequences of multiple forms of racism on development, we focus on the experiences of Black mothers and children relative to NLW counterparts (18, 33–38).

Racism may be particularly detrimental during early development, adversely impacting the socioemotional well-being of very young children (22). Hypothesized mechanisms center around the stress associated with parental experiences of racism and the resultant impact on parenting practices (39, 40). However, little is known about how the timing and duration of how exposure to racism might differentially impact children’s development and outcomes (33), including the extent to which Black women’s disparate experiences during the peripartum period impact the subsequent development of their children (41). Black mothers and children face significant challenges and disadvantages beginning in pregnancy (13) and continuing throughout all periods of early childhood (42, 43) with adverse effects on maternal mental health, parenting, and optimal child development (44). Much remains to be understood about the impact and mechanisms of systemic racism during early childhood. The dearth of robust, longitudinal studies limits our ability to develop culturally informed parenting interventions that can prevent the negative and lasting consequences of racism on early child development. With the emergence of the COVID-19 pandemic, studies need to consider the widening gap in Black maternal and child outcomes in light of a devastating new stressor that compounded existing inequities (45). No prior studies have collected data toward developing interventions that could mitigate syndemic effects. Thus, it is essential to examine the cascading effects of the syndemic to develop intervention targets, promote resilience, and combat vulnerability.

### Parents and parenting practices

Effective parenting techniques that promote optimal child development involve secure attachment (46), positive behavior support (47), an absence of harshness (48), and consistent positive reinforcement or non-violent discipline techniques (49). The rigor of prior parenting research is reflected in many worldwide policy directives encouraging the implementation of parenting interventions (50), with the most effective preventative interventions targeting early childhood (51). However, a host of individual and contextual factors shape parenting and the parent–child relationship. Both the bioecological system model (52) and family stress model (53) illustrate how multiple aspects of the home environment and parental resources, including parental psychopathology, familial wealth, neighborhood quality, and daily stressors, including racism, have a cascading impact on child development by shaping parenting practices (54, 55). For example, peripartum depression is linked to impaired mother–child attachment and bonding, atypical maternal facial expressions (56, 57), negative parent–child interactions (58–60), and poor child outcomes across cognitive, behavioral and emotional domains of functioning (61–63). Similarly, racism adversely impacts the infant-caregiver dyad and well-being of caregivers, leading to perturbations in the caregiver-infant attachment system, which can have lasting detrimental consequences for parenting practices (i.e., harsh and punitive), maternal mental health, and child outcomes (37, 64). Finally, there are sex differences in these associations, with evidence of greater vulnerability of males in early childhood, including poorer behavior regulation and stress reactivity during infancy and toddlerhood (65, 66), whereas girls are more susceptible to the negative effects of racial discrimination directed at caregivers at older ages (67). Thus, it is critical to support maternal mental health beginning in the prenatal period and to promote positive parenting practices across the first 5 years of life. In addition, it is important to investigate triad structures by including fathers/secondary caregivers as participants. A paucity of studies have investigated these pathways in the context of syndemic effects, which might differentially impact maternal mental health and, well-being, child mental health, and parenting practices for mothers and father/secondary caregivers and when comparing Black and NLW cohorts.

### The pandemic and mental health in parents and children

The COVID-19 pandemic created an unprecedented global health crisis, which was felt particularly acutely among families (68). Studies also documented increased rates of mental health problems, including anxiety, depression, and suicidality (69–71). Importantly, a disproportionate number of Black, Indigenous, and Latino/a/e/x parents were affected by job losses or reduced income relative to NLW parents (72). Data from our group, collected in April 2020, demonstrated that Black women had higher rates of depression and anxiety, and different COVID-19-specific worries when compared to NLW women (73). The lives of children were also negatively impacted by the COVID-19 pandemic (74). Similarly, more symptoms of posttraumatic stress disorder (PTSD) were reported among children isolated due to the H1N1 pandemic (75) and increases in aggression and PTSD symptoms were reported among children exposed to Hurricane Katrina (76). In particular, with physical distancing, families lost many social supports or other activities that would have otherwise promoted positive parent–child interactions (77), which may have been particularly detrimental to parents during the perinatal period, when social support is vital (78). Taken together, these differential experiences are concerning in light of robust evidence linking family stress and low parental social support to increased risk for disruptions in positive parenting (53). While data are still emerging, these findings together reflect the devastating impacts of the pandemic on parental and child mental health. The majority of existing data are from studies of adult populations (79) or samples of older children and adolescents (80), with a paucity of high-quality longitudinal evidence from studies that began following women in pregnancy and across the first 5 years of life.

### Objective

The COVID-19 pandemic represents a unique opportunity to (1) longitudinally characterize the effects of stress on the mental health of mothers and their children born at the start of the COVID-19 pandemic in the US (2); characterize syndemic effects on Black mothers and children compared to NLW peers; and (3) identify psychosocial mechanisms of the syndemic that shape early child development. The aim of this proposed project is to identify factors that impact early child development and establish critical early developmental periods that affect mental health trajectories. We address these goals by investigating the effect of the most catastrophic event of our lifetimes on women and children, coupled with careful characterization of the persistent trauma of racism and the syndemic experienced by Black families.

We outline here a study protocol for the *“Prenatal to Preschool”* study, which leverages an existing longitudinal cohort of racially diverse women, who were originally recruited while pregnant at the start of the COVID-19 pandemic. Our “Study Protocol” article details prospective research that does not have reportable data. We plan to utilize a novel, multi-method framework that incorporates (1): Diagnostic psychiatric interviews and questionnaires assessing mental health, syndemic effects, racism, and resilience, coupled with online surveys of fathers/secondary caregivers addressing similar themes (2); Structured mother–child interaction tasks and mother-and secondary caregiver/father-report questionnaires at two important developmental epochs (24 and 48 months) to evaluate child mental health, development, and parenting practices (3); Rich qualitative assessments of syndemic effects on Black women’s mental health at 36 months. Our project will establish the impact of COVID-19 pandemic stressors, racism, and endemic conditions on longitudinal mental health outcomes, while simultaneously identifying resiliency factors among Black and NLW mothers, fathers, and their children.

*H1*: Clinical Psychiatric Evaluation and Questionnaires.

We expect to establish differences in Black and NLW women in symptoms of psychopathology (including worries, stress, depression), resilience, and parenting. We will establish whether greater COVID-19 exposures and worries from prior waves (i.e., already collected Time 1 (T1)-Time 2 (T2)) are related to worse mental health outcomes (i.e., anxiety, stress, depression) at T4, and whether there are differences between Black and NLW women. We will also identify whether factors related to partner and social support, better emotion regulation, fewer syndemic effects, and higher self-reliance can mitigate the impact of COVID-19 pandemic stressors and lead to greater resilience and better mental health outcomes among both Black and NLW mothers and fathers/secondary caregivers.

*H2*: Parenting Practices and Questionnaires.

We expect to establish factors in the pregnancy and postpartum exposome that increase risk for disrupted development, particularly among Black children compared to NLW children. Importantly, we expect to identify that greater syndemic risk exposure and adverse mental health of parents will predict worse child longitudinal outcomes (i.e., symptoms of externalizing and internalizing psychopathology) at T4 and T5. However, we expect to establish that parental emotion regulation, positive parenting practices, fewer harsh parenting practices, and greater social and partner support, will be protective against adverse mental health outcomes in both Black and NLW children, leading to greater resilience.

*H3*: Qualitative Interview.

The expected outcomes are to identify syndemic factors that Black women defined as harmful to their experiences, as well as factors that mitigate these challenges. We hypothesize that Black women who report experiencing more racism in their everyday lives and throughout the pandemic, along with mistrust of the healthcare system/providers, will report more challenges. We plan to establish culturally informed intervention approaches to improve the accessibility, acceptability, and efficacy of interventions designed to improve the mental health outcomes of Black mothers and children.

## Methods and analysis

### Design

This proposed project follows mothers, fathers/secondary caregivers, and very young children through a longitudinal design (Table 1). There are three collection time-points along with data capture from mother and child medical records throughout. Review of mothers’ medical records included birth date, information related to birth and postpartum recovery, as well as medical and mental health history. Additionally, we leverage data already collected from the original cohort study at three time points: pregnancy (time 1; T1), 2–3 months postpartum (time 2; T2), and 12 months postpartum/infancy (time 3; T3).

**Table 1:** Research timeline, including previous study and current work.

	COVID-19 resilience in pregnancy and postpartum studies (previous study)			Prenatal to preschool			
	Time 1	Time 2	Time 3	Time 4	Time 5	Time 5	Time 6
Assessment	Prenatal Survey	2–4 months Postpartum Survey	12 months Postpartum Assessment & Survey	Maternal & Toddler Assessment; Mother & Father/Secondary Caregiver Surveys	SCID	Qualitative Interview	Preschool Assessment; Mother & Father/Secondary Caregiver Surveys
Method	Online	Online	Online and video “visit”	Online and video “visit”	Video “visit”	Video “visit”	Online and video “visit”
Child age	Gestational age, 8–39 weeks	9–15 weeks old	12–14 months old	26–32 months	36 months old	36 months old	48 months old

When children are age 24 and 48 months (time 4; T4 & time 6; T6), mothers will complete a battery of questionnaires and complete tasks with their child using toys are provided to the family. When the children are age 36 months (time 5; T5), mothers will complete the Structured Clinical Interview for DSM-5-TR (SCID-5-RV) (81). A subset including approximately 30–45 mothers will also be asked to complete a qualitative interview at T5. All study visits will be conducted virtually with rigorously created protocols. Fathers/secondary caregivers of the children enrolled in the study will be asked to complete questionnaires at T4 and T6. The Children’s Hospital of Philadelphia (Project number 22–019875) Institutional Review Board approved this study.

### Selection of participants

**Figure 1:**
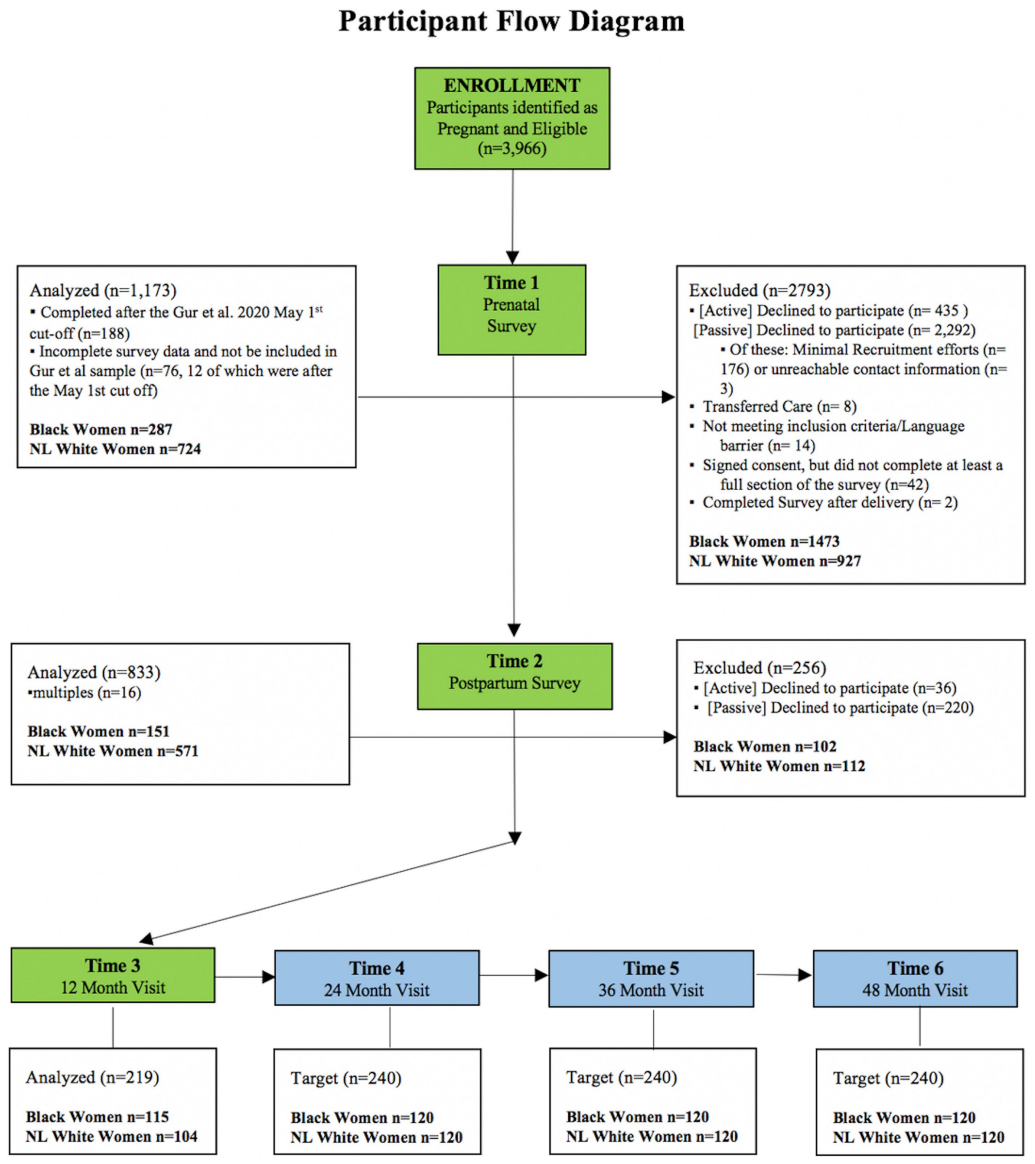
Consort diagram.

Enrolled mothers participated in the parent study: the COVID-19 Risk and Resilience Cohort of the IGNITE Program at the University of Pennsylvania and Children’s Hospital of Philadelphia. Participants were recruited from the Penn OBGYN outpatient clinics while pregnant during the early stages of COVID-19. The Penn Medicine Health System serves the majority of Philadelphia and delivers half of all newborns in the region, serving a unique population of racially diverse and historically underserved women, including 60% who are African American and 50% on Medicaid, making this a unique population to study racial disparities. The inclusion criteria for this project required all patients to be English-speaking. The exclusion criteria for this project included inability to complete the study procedures for any reason. Fathers/secondary caregivers were eligible to participate if the mother was eligible and provided their contact information. Mothers were permitted to select someone other than the father if the person was more involved in the child’s upbringing. Mothers who met all the inclusion criteria and none of the exclusion criteria were contacted to provide information about the study. If interested, they were consented and enrolled in the project. This mixed-method study with a quantitative screening and a qualitative interview employs purposive sampling methods with a focus on inclusion of participants from underrepresented communities. Inclusion and exclusion criteria are intended to enable generalizability and ensure data interpretability. Our planned sample for this proposed research is 240 mothers, fathers/secondary caregivers, and their children (Black, *n =* 120; NLW, *n =* 120) (Figure 1). 

### Measures and methods

To assess mental health outcomes as well as patterns of risk and resilience at 24 and 48 months, mothers will complete online questionnaires (via REDCap) (82, 83) targeting experiences arising from the COVID-19 pandemic and/or racial discrimination, social support, and other environmental factors. These will include those detailed below along with questions about COVID-19 worries and experiences (71, 84), demographics, income, and mental health care. Furthermore, we will leverage the data already collected from the COVID-19 Risk and Resilience Cohort, to see if early indicators of psychopathology existed and/or if symptoms worsened or improved. Fathers/secondary caregivers will complete a similar online survey allowing for exploration of partner support as a mitigating factor (Table 2).

**Table 2:** Methods and measures for questionnaires.

Construct	Measures	Variable in analysis	Method
Anxiety	GAD-7	Outcome and moderator	Mother and father/secondary caregiver report
Depression	BDI-II	Outcome and moderator	Mother and father/secondary caregiver report
Stress	PSS; PSI-SF; COVID worries	Predictor and moderator	Mother and father/secondary caregiver report
Social support	MSSI	Predictor and moderator	Mother report
Discrimination	EDS; Major Experiences of Discrimination Scale	Predictor and moderator	Mother and father/secondary caregiver report
Resilience	NSC; CD-RISC 10; MSSI	Predictor and moderator	Mother and father/secondary caregiver report
Mental health care	Seek help, make appt, type of provider, # of visits, etc.		Mother and father/secondary caregiver report
Medical care	WFPTS		Mother report
Home and caregiving environment	CECPAQ; CHAOS Scale; PBACE; AAPI; CRPR; APQ; PS	Predictor and moderator	Mother and father/secondary caregiver report
Infant development	ASQ:SE	Outcome	Mother and father/secondary caregiver report
Childhood development	CBCL	Outcome	Mother and father/secondary caregiver report

### Mother and father/secondary caregiver questionnaires (T4 and T6)

#### Perceived stress scale

The PSS scale assesses current stress levels (85). This scale exhibits high reliability and construct validity (86, 87). Parents are asked how often they have felt 10 aspects of stress in the last month using 5-point Likert scales ranging from “never” to “very often.”

#### Parenting stress index-short form

The PSI-4-SF is a 36-item scale version of the PSI, which identifies parent–child problem areas for children 1 month to 12 years of age, by asking if any significant life events have occurred in the past 12 months (88). The items are divided into three domains: Parental Distress, Parent–Child Dysfunctional Interaction, and Difficult Child (89). Scores from these three domains are combined to form a Total Stress scale, with scores ≥17 considered clinically significant.

#### Connor-Davidson resilience scale 10-item

The 10-item version of the CD-RISC assesses resiliency across a variety of parameters, including ability to adapt to change, cope with stress, and navigate adversity (90). Responses are on 0-to-4-point scale gaging how often the statements can be applied to the respondent (0 = not true at all to 4 = true nearly all the time). Scores from each item are combined to form a total score ranging from 0 to 40, with lower scores indicating greater resiliency.

#### Neighborhood safety and crime scale

The NSC is a 17-question survey asking respondents to indicate the degree to which issues are problematic in their neighborhood (e.g., vandalism, burglaries). Items are rated on a 3-point scale (0 = not a problem to 2 = a big problem). Responses are summed to create a total score where higher scores indicate greater issues in their neighborhood (91, 92).

#### Beck depression inventory, 2nd edition

Depression is assessed with the 21-item BDI-II that assesses depressive symptoms on a 0-to-4-point scale with scores >19 indicating moderate to severe depression (93).

#### Generalized anxiety disorder 7-item

The GAD-7 is a self-reported assessment for anxiety symptoms, aiming to quantify frequency over the preceding 2 weeks (94). Each question is rated on a 4-point scale (0 = not at all to 3 = nearly every day). Items are summed to create a total score where scores of 11 or higher indicate anxiety (95).

#### Maternal social support index

The MSSI is a 9-item questionnaire to assess aspects of a mother’s social support quantitatively and qualitatively (96). Questions relate to household and childcare related tasks. The two possible responses include “I take sole responsibility” or “Someone else does this task or helps me complete this task.” Items are summed to create an overall score where higher scores represent greater perceived maternal support.

#### Expanded everyday discrimination scale

The EDS is a 10-item scale used to measure subjective experiences of daily discrimination in minority populations (97). Items are rated on a 6-point scale (1 = almost every day to 6 = never). Scores are reverse coded and added together for a total score where higher scores reflect more frequency of discriminatory experiences (98).

#### Major experiences of discrimination scale 6-item version

The Major Experiences of Discrimination scale is a 6-item scale is used to record if significant discriminatory experiences have occurred across the respondent’s lifetime (99). Respondents reply “yes” or “no” to various experiences.

#### Confusion, hubbub, and order scale

Confusion, Hubbub, and Order Scale (CHAOS) is a 15-item parent report measure of the home environment with items rated on a 4-point scale (1 = not at all like your own home to 4 = very much like your own home), focusing on noise, confusion, clutter, and disorganization in the home environment (100).

#### Structured clinical interview for DSM-V

We will conduct an adapted version of the electronic SCID-V interviews with the entire cohort of mothers at child’s aged 36 months (T5) (101). Study analyze will describe responses at an item-level and scale-level, examine the proportion of mothers who would score “at-risk” based on standard scoring and diagnostic groupings, and examine whether there are differences in the proportion meeting diagnostic criteria for psychiatric disorders based on the following categorical variables: race, ethnicity, socioeconomic status and financial concerns, family structure and support, COVID-19 exposure, or educational attainment.

### Parenting practices

**Figure 2:**
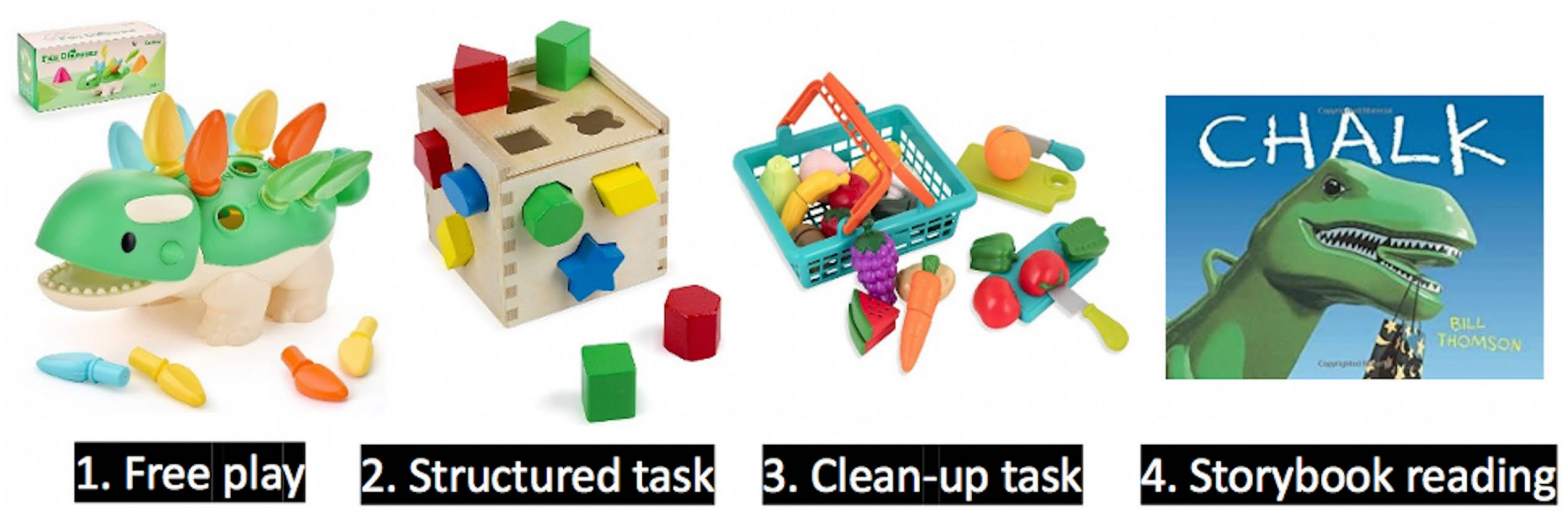
Task diagram for parenting practice assessment.

Children and their mothers will take part in two 30 to 45-min virtual visits (T4, age 2; T6, age 4). These virtual visits will be play games and complete semi-structured tasks together (e.g., free play, wordless storybook task). Task materials are mailed home in advance. All tasks are audio and video recorded using video meeting software (Zoom). Participants are asked to ensure that there are no distractions during these sessions. We use an adapted version of the Parent Child Interaction Rating System to code positive parental regard, parent stimulation of child cognitive development, parent positive reinforcement, child engagement, and parent negative regard (i.e., harshness) (102) (Figure 2).

### Mother and father/secondary caregiver-reported child mental health and developmental outcome measures (T4 and T6)

#### Child behavior checklist 1½-5

The CBCL assesses dimensions of psychiatric symptomatology, including anxiety, disruptive behavior, and emotional dysregulation (102). This scale has 99 items for quantitative evaluation and open-ended questions for qualitative information. Responses are on a 3-point scale reflecting how much the statement applies to the child in the preceding 2 months (0 = not true to 2 = very true or often true). Scores are summed within and across domains to provide problem scores with higher scores reflecting a parent’s greater reported presence and severity of symptoms in the child.

#### Ages and stages questionnaire

This questionnaire assesses overall child development, socio-emotional and language development when children are 2 and 4 years old (T4 and T6) (103). This scale includes 31 items with responses rated on a 3-point scale (0 = often or always, 5 = sometimes, and 10 = rarely or never, as well as an optional additional selection to check if the problem is a concern, scored with an additional 5 points). The total score is summed across all questions with scores <50 indicating no or low risk, 50–65 indicating monitor, and > 65 indicating need for referral. Two questions provide opportunities to write additional descriptions. There are three overall open response questions, two of which the respondent can indicate if there are problems, and detail, if applicable.

### Mother reported parenting practice measures (T4 and T6)

At T4 and T6, we will assess parenting via three questionnaires, with scores used to generate four core parenting constructs of harshness, warmth, emotion scaffolding, and positive control via established factor analytic approaches.

#### The preschool version of the Alabama parenting questionnaire

Thirty two items from the original APQ and assesses positive and negative aspects of parenting (104). The items are rated on 5-point Likert scales from 1 (never) to 5 (always). Ten items from the original APQ were not included due to inappropriate material for preschoolers.

#### Parents’ beliefs about children’s emotions questionnaire

A validated multi-faceted questionnaire, providing insight into mothers’ emotion-related socialization practices across 7 scales, including parental knowledge and control of children’s emotion. There are 16 items across these 7 scales (105).

#### The comprehensive early childhood parenting questionnaire

The CECPAQ evaluates parental behavior across five domains, however only two will be used for this study: Support and Positive Discipline (106). With this change, 17 items from the questionnaire on 6-point scales ranging from 1 (never) to 6 (always) to evaluate the domains of parenting presented above.

### Qualitative interviews

Interviews at T5 will elicit the heterogeneity of experiences and perspectives among Black postpartum women and gather specific feedback from mothers on the impact of the syndemic on their mental health, parenting practices, and child’s development. For example, mothers will be asked open-ended questions such as: “What was the most stressful about being pregnant during the pandemic?,” “Did you believe things were better or worse for you because of your race/ethnicity?,” “Did or do you have concerns about your healthcare providers or that of your child’s?” Questions will be designed to provide rich responses that can improve our understanding of the experiences, stressors, and support of Black mothers with children born during the COVID-19 pandemic. Importantly, interviews will explore whether Black women believe that the online surveys that our team, and other teams, have administered accurately capture race and ethnicity-specific differences. An advisory committee of three Black mothers will be formed to provide direct input on the questions asked of the subset of the main cohort, which will be 30–45 Black mothers. Finally, we will ask participants how best to improve and ensure inclusion of diverse communities in scientific research (e.g., via email, in-person, online recruitment), ask about barriers to participation they perceive, and specifically to identify the ways in which we, the researchers, can improve our methods and approaches to promote inclusivity. All qualitative interviews with mothers will be audio and/or videotaped and transcribed.

## Data analysis

### Parenting practices and child health

Analyzes will be conducted within a structural equation modeling (SEM) framework in Mplus version 8.2 using robust full information maximum likelihood (FIML) or weighted least squares means and variance (WLSMV) estimators as relevant (107). Model fit will be evaluated using established guidelines (108). We will use factor analytic techniques to create overarching multi-method factors and composite scores for child symptoms of externalizing and internalizing psychopathology at T4 and T6, which will be considered dependent variables in models. Using latent growth curve modeling, we will generate trajectories of maternal mental health, stress exposure, and social support over time (i.e., leveraging the fact that we have the same report measures across T1-T6) in relation to child externalizing and internalizing psychopathology (considered as correlated dependent variables). We will also use confirmatory factor analysis to generate parenting constructs that combine report and observed measures and use these scores to test conditional relations between study variables (e.g., stress x parenting) in relation to child mental health outcomes. Finally, we will test moderated relations between study variables, including race (i.e., Black vs. NLW children), as well testing moderation by sex as a biological variable in relation to all model pathways (e.g., parenting → child externalizing). Other model covariates, including all relevant demographic variables that were included in preliminary and published work using this sample (73), will be tested as exogenous predictors in the analyzes, modeling putative associations between exposure to stress/maternal mental health and child outcomes to account for any confounding influences (e.g., marital status, parity, age, child ASQ scores).

### Qualitative interview

We will review videotapes and audio recordings directly after each interview at T5 for an initial opportunity of analysis, which will be inductive allowing for the identification of common themes in our cohort in order to better characterize the varied responses and to identify emerging themes in their reports. The interviewer will review all transcripts for accuracy and will add notes about important non-verbal behaviors or characteristics of speech events (e.g., laughter, crying, nervous gestures). HIPAA-compliant transcription services will be used to convert raw field data to narrative transcripts. Raw data will be entered into a customized qualitative NVIVO software (109) database, a software package for qualitative analysis (QSR International). The NVIVO database will be used in order to store data, develop comprehensive coding schemas, code content, track emerging themes, and generate result summaries. The team will develop a coding structure and coding dictionary to be used in the analyzes. If differences in coding arise, the data will be discussed, and a consensus reached. We will use a modified grounded theory approach (110) to the analysis, consisting of systematic inductive guidelines for collecting and analyzing data building a framework to understand the interview data and build themes. This approach will allow descriptive analysis of the experiences reported by the mothers.

### Clinical assessment

Prior to analysis, we will evaluate the psychometric properties of questionnaire measures and compute descriptive statistics to examine distributions, with data transformations employed if warranted. Within Mplus vs. 8, we will use factor analytic techniques to create overarching factors and composite scores when multiple measures are used to assess a construct within time points, including for anxiety and depression (i.e., combining symptoms from SCID-V with self-report), stress, and social support. Latent Growth Curve (LGC) modeling will be used to test study aims, including modeling trajectories of mental health, stress and social support over time i.e., leveraging the fact that we will have the same report measures across T1-T4. To assess hypothesis 1, we will test unconditional LGC models to characterize the functional form of change in study variables over time and the covariance structure of residuals. There will be tests of conditional relations between study variables (e.g., between stress and mental health outcomes) and latent slope parameters, as well as moderated relations between study variables (e.g., by race, Black vs. NLW women.) Sub-hypotheses of hypothesis 1 will be probed following recommended guidelines (111, 112). Moreover, we will test putative interactions of social support, partner support, and resilience factors with stressors in relation to mental health outcomes, which will be probed following recommended guidelines (104, 105). Although we are interested in moderation by race, other model covariates, including all relevant demographic variables that were included in preliminary and published work using this sample (71), will also be tested as exogenous predictors in the analyzes modeling putative associations between exposure to stress and maternal mental health outcomes to account for any confounding influences (e.g., marital status, parity, age).

### Sample size

Monte Carlo studies were conducted to determine the power to evaluate study aims. Simulation studies included a population generating model of *N* = 200 (conservatively estimated to allow for attrition), assumed type 1 error rate of 0.05, and involved 5,000 replications (113, 114). The model for change used to express intercept and slope factors for each aim, when relevant, is expressed as: (level 1) [Y_ti_ = π_0i_ + π_1i_ (Time) + e_ti_] and (level 2) [π_0i_ = β_00_ + r_0i_] [π_1i_ = β_10_ + r_1i_]. Simulated data were generated under several constraints, including individual intercepts being drawn from a random, normal distribution, with specific means (β_00_) and standard deviations (r_0i_) guided by extant literature and preliminary evidence. The magnitudes of slope (β_10_) parameters were similarly informed. Errors at each time point (e_ti_) were modeled as conditionally independent and normally distributed. Monte Carlo studies were parameterized such that latent covariances and exogenous predictors exerted small-to-medium-sized effects on intercept and slope factors. Results parameterizing latent variables across units of analysis within timepoints or growth models within units of analysis across time suggest that, based on a total of 5,000 simulations using bootstrapped standard errors and 200 subjects, we can detect significant small (Cohen’s d ~ 0.38) (115) effects at a > 96% rate across all models.

### Discussion

Our innovative approach will allow us to identify pregnancy and early postpartum behavioral and environmental factors associated with neurodevelopmental and behavioral difficulties in children as young as 2 years old, who will be followed longitudinally. We will leverage already-collected survey data from participants who were recruited during the early phase of the pandemic and pandemic-related restrictions in the United States in April 2020 and have been followed successfully through three different time points. We will apply a novel, multi-method approach that includes psychiatric interviews with all the mothers at 36 months, in-depth observational paradigms of parent–child interactions to examine individual differences in child temperament and mental health at 24 and 48 months, as well as parenting practices, partner/secondary caregiver report, and detailed qualitative interviews with Black mothers at 36 months to characterize their syndemic experiences. Our approach will allow us to parse exposures in early development in ways that are tailored and personalized toward individual vulnerability and resilience, addressing the specific needs of racially diverse families and children. The goal is that the intended study will inform knowledge about the impact of the COVID-19 pandemic and other stressors, as well as the consequences of syndemic effects on Black mothers’ mental health and well-being.

We expect to establish factors in pregnancy and postpartum exposome that may increase the risk for disrupted development. Importantly, we expect to identify that greater syndemic risk exposure and adverse mental health of mothers will predict worse child longitudinal outcomes (i.e., symptoms of externalizing and internalizing psychopathology) at later time points. Furthermore, we expect to establish that maternal emotion regulation, more positive parenting practices, fewer harsh parenting practices, and greater social and partner support will be protective against adverse mental health outcomes in both Black and NLW children, leading to greater resilience.

Through the qualitative interviews, we expect to identify syndemic factors that Black women defined as harmful to their experiences, as well as factors that mitigate these challenges. We anticipate that Black women who report worse experiences of racism and mistrust of the healthcare system/providers will report more challenges. Combined with the expected outcomes above, we will utilize our findings to establish culturally informed intervention approaches to improve the accessibility, acceptability, and effectiveness of interventions designed to improve the mental health outcomes of Black mothers and children.

There are several expected limitations to the study. First, this is a longitudinal study and we anticipate some attrition in the sample. We hope to mitigate this limitation by ensuring that families have a positive experience during every interaction with our study team, providing generous compensation to families for their time, including remote participation with “home visit packs,” and overrecruiting. As Penn Medicine and CHOP are the major hospitals that serve our community, we also reduce participant burden by offering online assessments and clinical referrals for mothers and children as indicated. A related priority for this project and the research team is to recruit a diverse staff of Black, Indigenous, People of Color (BIPOC) to further aid with recruitment and retention. We will employ recruitment and retention protocols that are culturally-informed to foster trust between participants and research staff and value “meeting participants where they are,” maintaining contact between study visits, providing resources for referrals to other hospital-based or community services if needed, making accommodations for off-hours assessments, and in the current climate, creating remote virtual study visits. While our preliminary studies evidence our capacity to retain Black and NLW women during the postpartum period, a risk remains that we will fail to retain families for all of the proposed aims. We will send holiday and birthday cards as well as birthday gifts (e.g., t-shirts) to remind participants of their participation and build rapport. Second, we recognize that race is a complex social construct, with significant heterogeneity within racial groups. Penn Medicine asks patients to self-identify race and ethnicity, but we do not have access to genetic data for the purpose of ancestral mapping. Nonetheless, our study design addresses factors that exist at the community level, and membership in the social construct of race is what largely determines where a person lives, not their DNA. Third, we anticipate some missingness in our data. To address this issue, we will identify which variables are missing and compare women with and without these data available to capture what biases may be introduced by missingness that may not occur at random. Part of the “missingness” may be because some women will not answer all of the survey questions. As we have done in prior studies, we use robust full information maximum likelihood (FIML) procedures to account for up to 50% of missing data in the most robust way following recommended guidelines (116).

### Ethics and dissemination

The study presented was reviewed and approved by the Children’s Hospital of Philadelphia (Project Number 22–019875). The project was approved via cooperative agreement at the University of Pennsylvania. Consent was obtained from mothers for themselves and their child’s participation. Secondary caregiver/father consent was obtained from secondary caregivers or fathers for their participation as well. The consent process was conducted electronically via REDCap.

Historically underserved groups are disproportionately exposed and subjected to biases, prejudices, racism, and discrimination that lead to differential care starting very early in development (117, 118). We propose to advance the understanding of how such factors impact the mental health and well-being of Black women and their children through the timepoints of 24 months to 48 months with our multi-method study. Our approach addresses several weaknesses in the rigor of prior research (1); we propose diverse methods for quantifying multiple parameters of the exposome and behavioral indicators of aberrant behaviors and development in toddlers and preschoolers; and (2) we will improve knowledge about the experiences and stressors of those Black women most disproportionately impacted by healthcare inequities and racism.

The emergence of the COVID-19 pandemic made clear how little is known about the impact of multiple, overlapping, and systemic disparities on the experience of adversity in maternal mental health, and subsequent early child socioemotional, cognitive, and behavioral development. We leverage our established recruitment framework to enroll and retain Black women and children, seeking to balance racial disparities often found in the extant literature and improve our knowledge and care of diverse populations and those most disproportionately impacted by healthcare inequities. Across these multidisciplinary perspectives, our long-term goal is to identify areas of distress and resilience to better create targeted, culturally informed behavioral health interventions.

This proposed project was designed to investigate the syndemic effects on Black mothers and their children. Our findings will be disseminated through peer-reviewed publications, conference presentations, and lay reports. Given the multi-disciplinary team and multi-factorial nature of the research, efforts will be made to ensure dissemination is conducted through collaborative efforts, with focus given to those most knowledgeable on items included in this protocol. Above all, the authors of this “Study Protocol” article seek to promote racial equity and counter syndemic consequences for Black mothers and their developing children through engagement with the public debate on these issues.

## Ethics statement

The studies involving humans were approved by the Children’s Hospital of Philadelphia (Project number 22–019875). The studies were conducted in accordance with the local legislation and institutional requirements. Written informed consent for participation in this study was provided by the participants’ legal guardians/next of kin.

## Author contributions

WN: Conceptualization, Funding acquisition, Investigation, Methodology, Project administration, Resources, Supervision, Validation, Visualization, Writing – original draft, Writing – review & editing. TT: Investigation, Methodology, Methodology, Supervision, Visualization, Writing – original draft, Writing – review & editing. DE: Supervision, Visualization, Writing – original draft, Writing – review & editing. MH: Writing – review & editing. CA: Writing – review & editing. WH: Supervision, Writing – review & editing. KW: Investigation, Methodology, Project administration, Supervision, Writing – review & editing. AP: Data curation, Investigation, Methodology, Project administration, Writing – review & editing. KH: Writing – review & editing. YR: Writing – review & editing, Project administration, Supervision, Writing – review & editing. SK: Conceptualization, Funding acquisition, Methodology, Project administration, Supervision, Writing – original draft, Writing – original draft. FM: Conceptualization, Funding acquisition, Methodology, Project administration, Supervision, Writing – original draft, Writing – review & editing. XW: Data curation, Formal analysis, Software, Validation, Writing – review & editing. RG: Conceptualization, Funding acquisition, Methodology, Project administration, Supervision, Writing – original draft, Writing – review & editing. RW: Conceptualization, Funding acquisition, Methodology, Project administration, Supervision, Writing – original draft, Writing – review & editing.
